# Sensitive Detection of the Natural Killer Cell-Mediated Cytotoxicity of Anti-CD20 Antibodies and Its Impairment by B-Cell Receptor Pathway Inhibitors

**DOI:** 10.1155/2018/1023490

**Published:** 2018-03-19

**Authors:** Floyd Hassenrück, Eva Knödgen, Elisa Göckeritz, Safi Hasan Midda, Verena Vondey, Lars Neumann, Sylvia Herter, Christian Klein, Michael Hallek, Günter Krause

**Affiliations:** ^1^Department I of Internal Medicine, University of Cologne, Center of Integrated Oncology Cologne Bonn, Kerpener Str. 62, 50931 Köln, Germany; ^2^Cologne Cluster of Excellence on Cellular Stress Responses in Aging-Associated Diseases (CECAD Cologne), Cologne, Germany; ^3^Roche Innovation Center Zurich, Pharma Research and Early Development (pRED), Schlieren, Switzerland

## Abstract

The antibody-dependent cell-mediated cytotoxicity (ADCC) of the anti-CD20 monoclonal antibodies (mAbs) rituximab and obinutuzumab against the cell line Raji and isolated CLL cells and its potential impairment by kinase inhibitors (KI) was determined via lactate dehydrogenase release or calcein retention, respectively, using genetically modified NK92 cells expressing CD16-176V as effector cells. Compared to peripheral blood mononuclear cells, recombinant effector cell lines showed substantial alloreactivity-related cytotoxicity without addition of mAbs but afforded determination of ADCC with reduced interassay variability. The cytotoxicity owing to alloreactivity was less susceptible to interference by KI than the ADCC of anti-CD20 mAbs, which was markedly diminished by ibrutinib, but not by idelalisib. Compared to rituximab, the ADCC of obinutuzumab against primary CLL cells showed approximately 30% higher efficacy and less interference with KI. Irreversible BTK inhibitors at a clinically relevant concentration of 1 *μ*M only weakly impaired the ADCC of anti-CD20 mAbs, with less influence in combinations with obinutuzumab than with rituximab and by acalabrutinib than by ibrutinib or tirabrutinib. In summary, NK cell line-based assays permitted the sensitive detection of ADCC of therapeutic anti-CD20 mAbs against CLL cells and of the interference of KI with this important killing mechanism.

## 1. Introduction

Monoclonal antibodies (mAbs) play an important role in the treatment of chronic lymphocytic leukemia (CLL) and other B-cell malignancies. Thus the anti-CD20 mAb rituximab is part of the current standard chemoimmunotherapeutic regimen for first-line treatment of CLL patients without comorbidity [1] and the benefit for CLL patients with comorbidity under treatment with the DNA damaging agent chlorambucil was superior in combination with the glycoengineered type II anti-CD20 mAb obinutuzumab than with rituximab [2]. The management of CLL has undergone profound changes also owing to the availability of novel agents that target B-cell receptor signaling [3]. Among these, the kinase inhibitors (KI), idelalisib and ibrutinib that target PI3K-*δ* and BTK, respectively, have gained approval for the treatment of CLL [4, 5]. This clinical progress was supported by preclinical drug assessment for selecting development candidates and recognizing the underlying mechanisms. For further improvement of therapeutic options these preclinical efforts need to be continued, for example, for designing efficacious drug combinations.

Cell killing by therapeutic mAbs proceeds via direct cell death induction and via indirect mechanisms that are mediated by the Fc (fragment crystallizable) portion of mAbs and include complement-dependent cytotoxicity (CDC) as well as antibody-dependent cell-mediated cytotoxicity and phagocytosis (ADCC and ADCP) [6]. Effector cells expressing activating Fc*γ* receptors (Fc*γ*Rs), for example, CD16, are activated by binding the Fc portions of antibodies once these are in contact with antigen and subsequently release cytotoxic agents such as perforin and granzymes that accomplish target cell lysis. Thus, in ADCC, the specificity of mAbs provided by the adaptive immune system is linked to powerful innate immune effector functions by binding of Fc regions to Fc*γ* receptors, for example, on NK cells.* In vitro* assays of ADCC can be performed in a variety of formats employing different effector cells and a wide range of direct and indirect detection methods [6].

As a type II anti-CD20 mAb obinutuzumab has a substantially different binding mode to CD20 as rituximab and enhanced direct cytotoxicity and Fc-mediated functions [7]. For obinutuzumab as a single agent we have previously shown more potent CLL cell depletion from whole blood samples and stronger direct cytotoxicity against CLL cells than by rituximab [8]. In addition the mechanisms of obinutuzumab have been extensively compared with other anti-CD20 mAbs and characterized with regard to the effects of glycoengineering on ADCC and ADCP [9, 10].

Owing to independent mechanisms of action, mAbs are considered as promising combination partners of KI, however, with the possible risk of interference of kinase inhibition with major mechanisms of action of mAbs, for instance, ADCC. The irreversible BTK inhibitor ibrutinib, however, was found to antagonize the ADCC of rituximab [11], while in the presence of the phosphatidylinositide-3-kinases- (PI3K-) *δ* inhibitor idelalisib that of alemtuzumab was maintained [12].

The goal of the present study was to combine the use of (1) nonradioactive ADCC detection, (2) NK92-derived recombinant effector cell lines [13, 14], and (3) primary CLL samples as target cells in nonautologous assays. With NK92 cell line-based assays, we were able to distinguish the ADCC of rituximab and obinutuzumab and to evaluate the interference of kinase inhibitors with the ADCC of these anti-CD20 mAbs.

## 2. Materials and Methods

### 2.1. Cell Lines and Patient Samples

The CLL-derived EBV-transformed lymphoblastoid lines JVM-3 and Mec1 as well as the Burkitt lymphoma cell line Raji were purchased from the German collection of microorganisms and cell cultures (DSMZ, Braunschweig, Germany) and used as target cells in ADCC assays. Primary CLL cells for use as target cells were isolated from peripheral blood samples from patients who were previously diagnosed for CLL according to standard criteria. Blood samples were obtained with informed consent in accordance with the World Medical Association Declaration of Helsinki following a study protocol approved by the local ethics committee at the University of Cologne (approval number 11-319).

Recombinant NK92-derived effector cell lines had been engineered to express the high affinity allele of the Fc*γ*R IIIa, also known as CD16-158V or 176V depending on exclusion or inclusion of the signal peptide in the amino acid count, and were obtained under material transfer agreements with Conkwest or Roche, respectively. The cell lines CD16.NK92.26.5 [13] and NK92-1708-CD16 clone LC3E11 [15] in this report are referred to in abbreviated form as 26.5 and 1708-LC3E11, respectively. NK92 cells as obtainable from the American Type Culture Collection do not express Fc*γ*R IIIa. The derivative cell lines used here were not only genetically modified to express CD16 but also involve subclones with altered expression of killer cell Ig-like receptors (KIRs) that leads to dampened alloreactivity in ADCC assays with nonautologous hematopoietic cells.

### 2.2. Therapeutic Antibodies and Small Molecule Drugs

Obinutuzumab was a kind gift from Roche Glycart. Rituximab and alemtuzumab were obtained from the hospital pharmacy. Antibodies were used at a concentration of 10 *μ*g/ml that is known to elicit maximal effects. The PI3K inhibitors idelalisib, duvelisib (IPI-145), and copanlisib (BAY 80-6946) as well as the irreversible BTK inhibitors ibrutinib, acalabrutinib (ACP-196), and tirabrutinib (ONO/GS-4059) were purchased from Selleck via AbSource (Munich, Germany) and used as stock solutions prepared in DMSO. The DMSO concentration in cell culture media was limited to 0.5%.

### 2.3. Cell Isolation and Culture

For isolation of CLL cells, Ficoll-Paque Plus sedimentation (GE Healthcare, Freiburg, Germany) was preceded by incubation of whole blood with the Rosette Sep B-cell purification antibody cocktail (Stem Cell Technologies) to aggregate unwanted cells with erythrocytes. The purity of isolated CLL cells was determined by flow cytometry using FITC-labeled anti-CD5 and PE-labeled anti-CD19 antibodies (BD Biosciences, Heidelberg). Isolated CLL cells and cell lines used as target cells were cultured in RPMI medium supplemented with 10% heat-inactivated fetal calf serum (FCS) and antibiotics and antimycotics (Gibco, Thermo Fisher Scientific, Darmstadt, Germany) at 37°C in a humidified atmosphere containing 5% carbon dioxide.

For use as effector cells in ADCC assays, peripheral blood mononuclear cells (PBMCs) from a healthy donor were isolated from heparinized blood samples by Ficoll gradient centrifugation. NK92-derived recombinant cell lines were cultured in *α*-MEM without nucleosides (Life Tech) supplemented with 10% heat-inactivated FCS, 10% heat-inactivated horse serum, 0.1 mM *β*-mercaptoethanol, 1.5 mM L-glutamine, 0.2 mM myoinositol, 1 mM sodium pyruvate, and 2 *μ*M folic acid. 26.5 cells were supplemented with 100 U/ml IL-2 (Immunotools) upon each splitting and controlled for GFP expression. 1708-LC3E11 cells were IL-2-independent and selected with 5 *μ*g/ml puromycin.

### 2.4. Determination of Antibody-Dependent Cell-Mediated Cytotoxicity (ADCC)

Cocultures of target and effector cells were performed on U-bottom 96-well plates in AIM-V medium (Thermo Fisher Scientific). Target cells, that is, Raji cells and primary CLL cells, were seeded at a cell density of 3 × 10^4^ cells per well, with the exception of JVM-3 and Mec1 cells, the density of which was 1.5 × 10^5^ per well. The excess of effector over target cells was 5- or 15-fold with NK92-derived recombinant effector cell lines or freshly isolated PBMCs, respectively. After coincubation with effector cells, target cell lysis was measured with an LDH release cytotoxicity detection kit according to the instructions of the supplier (Roche Diagnostics, Mannheim, Germany). LDH released from lysed cells leads to reduction of iodo tetrazolium chloride. The amounts of the colored formazane formed were measured via absorption at 450 nm in a FluoSTAR OPTIMA plate reader. First effector cells were plated, followed by target cells and finally 10 *μ*g/ml of mAbs. Low and high controls, the spontaneous or maximal LDH release, respectively, were determined from target cells alone or after complete lysis of target plus effector cells by 1% Triton X-100. Cocultures of target and effector cells were performed without or with addition of antibody. ADCC was calculated as the antibody-dependent enhancement of cytotoxicity in cocultures of target and effector cells.

With primary CLL cells as target cells cytotoxicity was detected via calcein retention instead of LDH release. For this purpose, CLL cells were stained for 30 minutes with 3.5 *μ*M calcein-AM (Promokine) and washed three times in AIM-V medium before coculture with effector cells. For determining calcein retention in specifically labeled target cells, the cells remaining in cocultures were sedimented by centrifugation for 5 min at 400 ×g and lysed in 200 *μ*l of 5 mM sodium borate buffer containing 1% Triton X-100. After transfer of 180 *μ*l of the lysates into black 96-well plates (Nunc) fluorescence signals were measured with excitation and emission wavelengths of 485 and 520 nm, respectively, in a FluoSTAR OPTIMA plate reader. The calcein retention in target cells without addition of effector cells, corresponding to spontaneous label release, was set to 100% and that in completely lysed cocultures to 0%. The calcein retention values were converted to the reciprocal percentages of cytotoxicity to comply with LDH release measurements and commonly used presentation of ADCC assay results.

### 2.5. Data Presentation and Statistics

In box plot diagrams, boxes represent the middle quartiles of distributions and whiskers the maximal and minimal ranges, which are limited to 1.5 interquartile ranges. Outliers are indicated by filled diamonds, while plus signs denote arithmetic means.

Significance levels were determined by two-tailed, paired Student's* t*-test and indicated as n.s.: not significant, ^*∗*^*p* < 0.05; ^*∗∗*^*p* < 0.01; ^*∗∗∗*^*p* < 0.001.

## 3. Results

### 3.1. Measuring ADCC with Different Effector Cells

**Figure 1:**
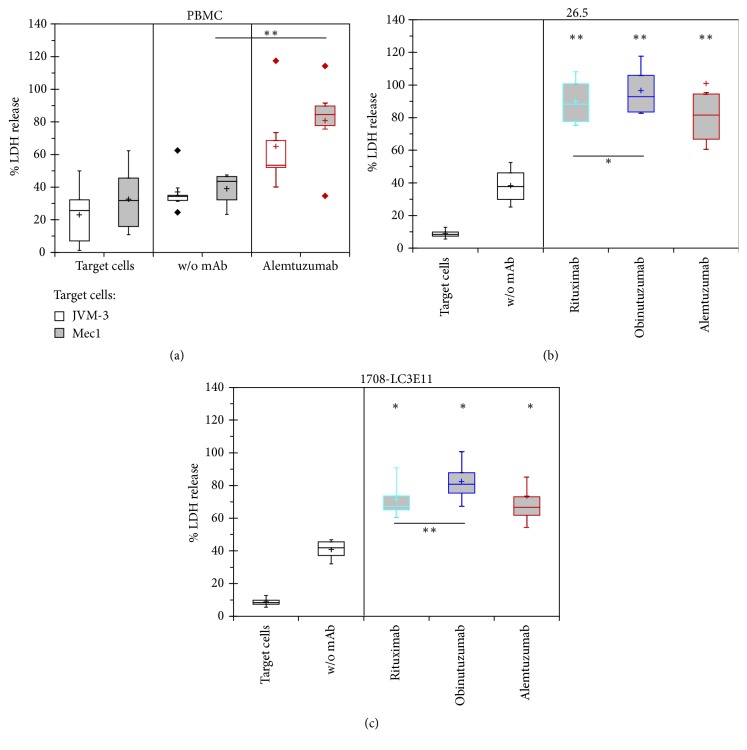
*ADCC mediated by PBMCs and NK92-derived cell lines*. Percentages of maximal LDH release from target cells were determined with either PBMCs at 15-fold excess (a) or of two strains of genetically modified NK92 cells expressing CD16-176V at 5-fold excess (b, c) with or without 10 *μ*g/ml of the indicated antibodies. (a) In six independent experiments 1.5*∗*10^5^ JVM-3 or Mec1 target cells per well were incubated with PBMCs from a healthy donor with or without alemtuzumab for 4 hours. (b, c) In four independent assays each, LDH release from 3*∗*10^4^ Raji cells was determined after 2 hours of coculture with the effector cell lines 26.5 or 1708-LC3E11 in the absence or presence of three different antibodies. LDH release from mixtures of effector and target cells without or with antibodies was compared by paired Student's* t*-test. In addition, the efficacy of the ADCC elicited by rituximab versus obinutuzumab was compared by paired* t*-test.^*∗*^*p* < 0.05; ^*∗∗*^*p* < 0.01.

NK92-derived effector cell lines were compared to unstimulated PBMCs in an assay format that uses LDH release from target cells as a measure of cytotoxicity (Figure 1). Along with spontaneous LDH release from target cells alone, that from cocultures of target and effector cells was monitored as background for the determination of the enhancement of cytotoxicity by addition of mAbs, which were used at a concentration of 10 *μ*g/ml assuring maximal response.

Compared to spontaneous target cell lysis, the relative LDH release was significantly increased by approximately 30% in the presence of effector cell lines (Figures 1(b) and 1(c)), but only marginally, that is, by less than half of that amount, in cocultures with PBMCs (Figure 1(a)). Despite different target cell lines, cell densities, and incubation times, the substantial antibody-independent cytotoxicity in cocultures with target cells appears to be connected with alloreactivity compared to that in those with donor-derived effector cells and owing to its size it needs to be carefully separated from the antibody-dependent increase of cytotoxicity that defines ADCC in the proper sense. In this context it may be worthwhile noting that NK92 cells, which had been engineered only for forced CD16 expression, but not for expression of novel KIRs, are functional for ADCC assays with nonhematological target cells [16] but yielded high spontaneous antibody-independent cytotoxicity owing to alloreactivity in cocultures with Raji cells that surpassed and masked ADCC (not shown).

Despite the higher cytotoxicity observed in cocultures of target cells with NK92-derived effector cell lines than with PBMCs, alemtuzumab additionally induced equal or higher ADCC in the cell line-based assays. Moreover the ADCC of alemtuzumab observed with PBMCs as effector cells was higher against Mec1 than JVM-3 cells. Comparing two similar NK92-derived effector cell lines, 26.5 cells indicated higher percentages of cytotoxicity altogether and especially a greater increment of cytotoxicity upon addition of mAbs than 1708-LC3E11 cells, whereas the efficacy of the ADCC of obinutuzumab appeared 13% or 36% higher than that of rituximab with 26.5 or 1708-LC3E11, respectively. In summary, the range of the variance between individual experiments was higher with PBMCs as effector cells as evidenced by higher frequency of outliers than with recombinant NK92 cells. Consequently the investigated effector cell lines afford more sensitive detection of ADCC and its impairment by kinase inhibitors than PBMCs as effector cells.

### 3.2. Interference of Kinase Inhibitors with the ADCC of Anti-CD20 mAbs against Raji Cells

**Figure 2:**
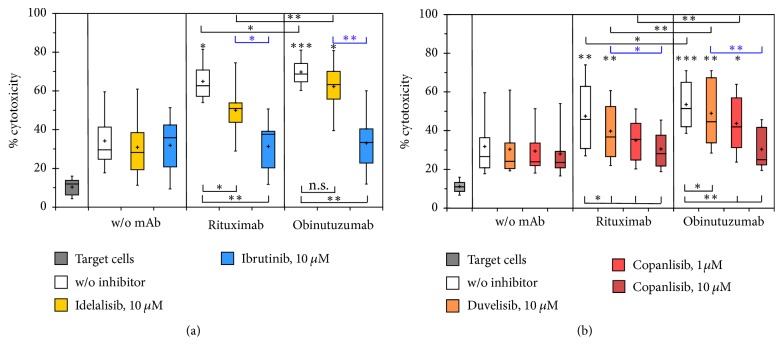
*Impairment of ADCC against Raji cells by kinase inhibitors*. The antibody-dependent increase in the percentages of maximal LDH release was determined with 5-fold excess of 26.5 effector cells in the absence or presence of 10 *μ*M idelalisib or ibrutinib (a) or the PI3K inhibitors duvelisib and copanlisib (b). Data were derived from nine or eight independent experiments, each of which was performed with triplicate samples, in (a) and (b), respectively. Asterisks above the boxes and whiskers denote the significance of enhanced cytotoxicity compared to the control without addition of antibodies and inhibitors as determined by paired* t*-tests, while the *p* value ranges for the impairment of ADCC compared to anti-CD20 antibodies as single agents are indicated beneath. Furthermore comparisons by paired* t*-test were performed among mAb and KI treatments and indicated at the top of the diagrams in black and blue print, respectively. ^*∗*^*p* < 0.05; ^*∗∗*^*p* < 0.01; ^*∗∗∗*^*p* < 0.001.

Currently two kinase inhibitors have gained approval for the treatment of CLL and other B-cell malignancies, namely, the PI3K-*δ* inhibitor idelalisib and the irreversible BTK inhibitor ibrutinib. Using 26.5 cells as effector cells we determined the ADCC against Raji cells of combinations of CD20 antibodies with these two targeted agents via LDH release (Figure 2(a)). To challenge the stability of NK cell-mediated ADCC, both kinase inhibitors were used at a concentration of 10 *μ*M that clearly surpasses clinically achievable concentrations.

The LDH release owing to alloreactivity in cocultures of NK-92-26.5-CD16 effector cells and Raji target cells was hardly influenced by these high concentrations of idelalisib and ibrutinib. The significant enhancement of cytotoxicity due to addition of anti-CD20 mAbs was largely maintained in the presence of 10 *μ*M idelalisib, indeed more effectively in the combinations with obinutuzumab than with rituximab. In contrast, the ADCC of CD20 antibodies was virtually abolished by 10 *μ*M ibrutinib. Consequently the cytotoxicity against Raji cells of rituximab and obinutuzumab in combination with idelalisib was significantly higher than in combination with ibrutinib (*p* = 0.009 and *p* = 0.007, resp., for *n* = 9).

The widely different impairment of ADCC by idelalisib and ibrutinib prompted us to test whether further PI3K inhibitors in development as drugs against B-cell malignancies, namely, duvelisib and copanlisib, behaved similarly as idelalisib in the same assay system (Figure 2(b)). In combinations with rituximab and obinutuzumab also these inhibitors were used at a concentration of 10 *μ*M, which by far exceeds clinically obtainable concentrations, and, in the case of copanlisib additionally at 1 *μ*M, owing to previously noted higher cytotoxicity against malignant B-cells of copanlisib compared to idelalisib [17]. Like the other investigated BCR signaling inhibitors also these PI3K inhibitors did not substantially affect the background cytotoxicity in mixtures of effector and target cells without addition of mAbs. The ADCC against Raji cells mediated by anti-CD20 mAbs was influenced by duvelisib in a similar manner as by idelalisib; that is, ADCC was only marginally impaired, with slightly stronger reduction of rituximab than of obinutuzumab effects. Copanlisib concentration-dependently led to a considerably stronger impairment of ADCC than idelalisib and duvelisib. With 10 *μ*M copanlisib the enhancement of LDH release owing to addition of mAbs was almost completely eliminated. In six assay repetitions that contained all four investigated KI the impairment of ADCC by idelalisib and duvelisib appeared approximately equal, but stronger by ibrutinib than by copanlisib. Significant ADCC of anti-CD20 mAbs was maintained in the presence of 10 *μ*M duvelisib and in the case of obinutuzumab also in combination with 1 *μ*M copanlisib.

In summary, high concentrations of kinase inhibitors did not interfere with the background LDH release from mixtures of Raji target cells with NK92-26.5 cells but impaired the ADCC mediated by anti-CD20 mAbs to different degrees. ADCC was significantly disturbed by the BTK inhibitor ibrutinib and the pan-class I PI3K inhibitor copanlisib, in contrast to the PI3K inhibitors idelalisib and duvelisib that selectively target the PI3K-*δ* isoform.

### 3.3. ADCC against CLL Cells of Rituximab and Obinutuzumab in Combination with Kinase Inhibitors

**Figure 3:**
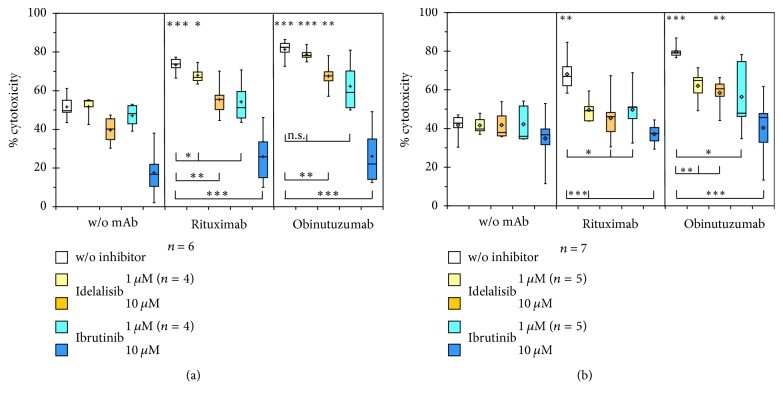
*Impairment of ADCC against primary CLL cells by kinase inhibitors*. The antibody-dependent increase in the percentages of minimal calcein retention was determined in six or seven independent experiments with 3-fold excess of recombinant 26.5 (a) or 1708-LC3E11 (b) effector cells over CLL target cells. Cytotoxicity was compared to controls without antibodies by paired two-tailed* t*-test. ^*∗*^*p* < 0.05; ^*∗∗*^*p* < 0.01; ^*∗∗∗*^*p* < 0.001.

For ADCC measurements in clinical samples, isolated CLL cells were used as target cells instead of the cell line Raji in the newly established ADCC assay using 26.5 or 1708-LC3E11 effector cells. Even at a three times higher target cell density as used with Raji cells, that is, 9 × 10^4^ cells per well, total lysis of CLL cells by Triton X-100 did not yield sizable LDH release (not shown). Therefore measurements of ADCC against primary CLL samples were performed after labeling these target cells with calcein for fluorimetric determination of label release during coculture with effector cells. The ADCC of anti-CD20 mAbs against CLL cells was determined in combinations with idelalisib or ibrutinib at concentrations of 10 *μ*M as used in the determinations of LDH release from Raji cells and of 1 *μ*M to approach clinically relevant concentrations (Figure 3).

Similarly, for the ADCC against Raji cells detected via LDH release (Figures 1(b) and 1(c)) also using isolated CLL cells as target cells with detection of calcein release, rituximab and obinutuzumab elicited 20–40% higher cytotoxicity against target cells than in cocultures without addition of mAbs. Ibrutinib impaired the NK92-26.5-mediated ADCC of rituximab or obinutuzumab against CLL cells more strongly than idelalisib but also affected the cytotoxicity against mixtures of target and effector cells (Figure 3(a)), in a similar or divergent manner, respectively, as against Raji cells (Figure 2). The fluorescence signals in target cells in the absence of effector cells, which corresponded to spontaneous calcein retention and were set as maximal values, were equal without or with treatment with KI (not shown). Unlike the ADCC of anti-CD20 mAbs against Raji cells, that against isolated CLL cells was impaired by 10 *μ*M idelalisib, although with significantly maintained ADCC of obinutuzumab. 1 *μ*M ibrutinib decreased the ADCC of the assessed anti-CD20 mAbs to a similar degree as 10 *μ*M idelalisib. Overall, cytotoxicity appeared to be impaired more strongly by kinase inhibitors in cocultures with CLL than with Raji target cells.

Comparing the two NK92-derived effector cell lines, the alloreactivity-related antibody-independent cytotoxicity was less affected by 10 *μ*M ibrutinib in cocultures with 1708-LC3E11 than with 26.5 cells. In addition 1 *μ*M idelalisib interfered more strongly with the ADCC of anti-CD20 mAbs mediated by 1708-LC3E11 compared to 26.5 effector cells. The ADCC of rituximab and obinutuzumab was impaired by idelalisib and ibrutinib with greater significance in assays using 1708-LC3E11 than 26.5 effector cells.

**Figure 4:**
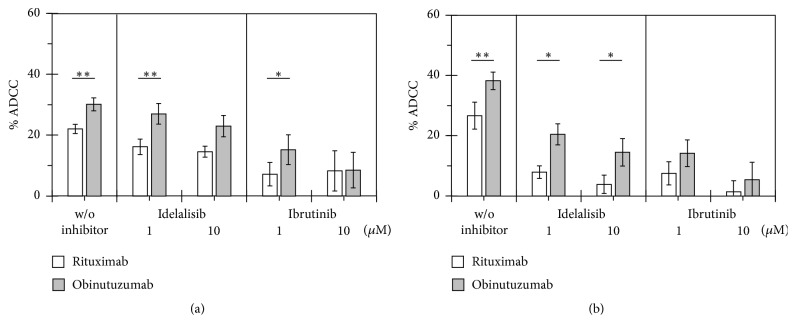
*Detailed analysis of the impact of idelalisib and ibrutinib on the ADCC of rituximab and obinutuzumab*. The differences in the cytotoxicity against CLL cells in cocultures with the NK92-derived effector cells 26.5 (a) or 1708-LC3E11 (b) in the presence and absence of rituximab and obinutuzumab were calculated from the data shown in Figure 3. Means and standard errors of the means are shown. The ADCC of rituximab and obinutuzumab was compared by paired two-tailed* t*-test. ^*∗*^*p* < 0.05; ^*∗∗*^*p* < 0.01.

For a more detailed analysis of the impact of KI on ADCC, the percentages of cytotoxicity in the absence of rituximab and obinutuzumab were subtracted from those in their presence and compared head to head (Figure 4). The impairment of both ADCC by KI and the superior ADCC of obinutuzumab compared to rituximab was indicated more clearly by 1708-LC3E11 effector cells (Figure 4(b)) than by 26.5 cells (Figure 4(a)). Obinutuzumab consistently showed significantly higher efficacy of ADCC than rituximab. This difference in ADCC efficacy was largely maintained in the presence of idelalisib and with 1 *μ*M ibrutinib, which means less impaired ADCC of obinutuzumab in the presence of KI compared to rituximab.

### 3.4. Impact of BTK Inhibitors on the ADCC against CLL Cells of Rituximab and Obinutuzumab

**Figure 5:**
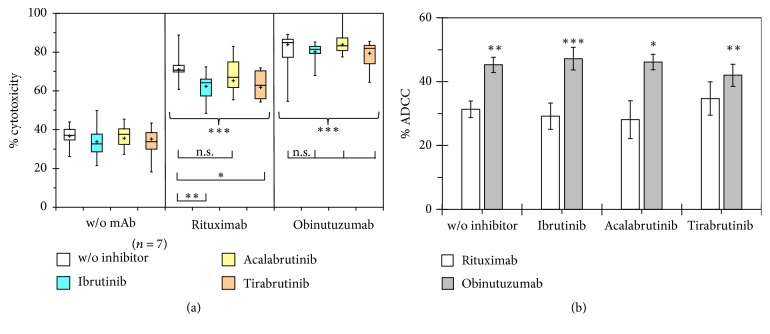
*Comparison of the impairment of ADCC against primary CLL cells by different irreversible BTK inhibitors*. The antibody-dependent increase in the percentages of minimal calcein retention was determined with 3-fold excess of 1708-LC3E11 effector cells over CLL target cells in seven independent experiments. (a) Asterisks above the boxes and whiskers denote the significance of enhanced cytotoxicity compared to the control without addition of antibodies and inhibitors as determined by paired* t*-tests. Furthermore combination treatment was compared to that with anti-CD20 antibodies as single agents. (b) The mean differences ± SEM in the cytotoxicity against CLL cells in the presence and absence of rituximab and obinutuzumab were calculated from the data shown in (a). The ADCC of rituximab and obinutuzumab was compared by paired two-tailed* t*-test. ^*∗*^*p* < 0.05; ^*∗∗*^*p* < 0.01; ^*∗∗∗*^*p* < 0.001.

1708-LC3E11 effector cells, which had indicated the impairment of ADCC against CLL cells by KI with greater sensitivity than 26.5 cells, were employed to compare ibrutinib with the second-generation irreversible BTK inhibitors acalabrutinib (ACP-196) and tirabrutinib (GS-4059) at a clinically relevant concentration of 1 *μ*M (Figure 5(a)). With a different set of CLL samples than that used in Figure 4(b), rituximab and obinutuzumab significantly enhanced NK cell-mediated cytotoxicity by 31% and 45%, respectively, and the significance of ADCC, which is equivalent to this enhancement, was maintained in combination with the investigated irreversible BTK inhibitors at clinically relevant concentrations of 1 *μ*M. Significant impairment of ADCC was observed in combinations of rituximab with ibrutinib and tirabrutinib but not acalabrutinib and did not occur in the tested combinations with obinutuzumab. Of note, acalabrutinib with improved BTK selectivity led to weaker impairment of ADCC than ibrutinib and tirabrutinib. Obinutuzumab showed 44% higher efficacy of ADCC than rituximab (Figure 5(b)). This difference was even augmented to approximately 60% in combinations of anti-CD20 mAbs with ibrutinib and acalabrutinib, since the ADCC of obinutuzumab was less affected than that of rituximab also by second-generation BTK inhibitors.

## 4. Discussion

Convenient and robust NK cell line-based assays permitted the sensitive detection of ADCC of therapeutic anti-CD20 antibodies against CLL cells and of the interference of KI with this important killing mechanism of mAbs. To our knowledge, in the present report, the use of recombinant NK92-derived effector cell lines expressing CD16 is combined for the first time with that of primary CLL samples as target cells for nonradioactive ADCC determination.

Compared to spontaneous target cell lysis, the antibody-independent cytotoxicity in cocultures with effector cell lines was substantially increased, in contrast to cocultures with PBMCs. This is in agreement with the expectation that clonal NK cell lines may elicit stronger alloreactivity than natural NK cell populations that achieve tolerance by expressing various different combinations of activating and inhibitory KIRs. Compared to PBMCs as effector cells, the interassay variability was reduced with NK92-derived effector cell lines in a similar manner as in assays using purified NK cells [18].

The ADCC against Raji cells elicited by rituximab was only approximately one-third of the background lysis with NK92 cells expressing CD16 that had not been modified for altered KIR expression [19], but slightly higher than the corresponding spontaneous cytotoxicity with 26.5 effector cells. The observed still comparatively high antibody-independent cytotoxicity in cocultures with the recombinant NK92-derived cell lines expressing CD16 at hand emphasizes the need to take it into account as a contribution to the overall cytotoxicity observed in the presence of both antibodies and effector cells. For the calculation of ADCC in the literal sense, the appropriate background to be subtracted therefore is the alloreactivity-related cytotoxicity in cocultures of target and effector cells and not spontaneous lysis of target cells alone. Although the percentages of cytotoxicity obtained by Duong et al., 2015 [20], with NK92-26.5 cells expressing CD16 as effector cells are similar to the overall cytotoxicity in the presence of mAbs as observed here, they are lacking the important control of target and effector cells without mAbs and do not allow the distinction of KI effects on antibody-dependent or independent cytotoxicity. Using similar NK92-derived effector cells we could show that all investigated inhibitors of BTK and PI3K-*δ* at clinically relevant concentrations exclusively affected ADCC and left the alloreactivity-related antibody-independent cytotoxicity undisturbed. Only at a 10-fold higher concentration, ibrutinib also inhibited the NK cell-mediated antibody-independent cytotoxicity of anti-CD20 mAbs.

Our observation that the ADCC of anti-CD20 mAbs was inhibited more severely by the irreversible BTK inhibitor ibrutinib than by the PI3K-*δ* inhibitor idelalisib is in agreement with the expectation from observations with these inhibitors separately [11, 12, 17] and with other direct comparisons of the impact of these inhibitors on ADCC in different formats, namely, NK cell-mediated ADCC detected via LDH or ^51^Cr release, respectively, [21, 22] or in an NK effector cell line-based assay with detection by calcein release [20]. Following up on the preclinical assessment of selected PI3K inhibitors [17], duvelisib, similar to idelalisib, hardly disturbed NK cell mediated ADCC, in line with the very similar molecular structure and in spite of targeting PI3K-*γ* in addition to PI3K-*δ*. In contrast, copanlisib, which was recently approved for treatment of follicular lymphoma, strongly impaired the ADCC of rituximab and obinutuzumab at a concentration of 10 *μ*M but permitted significant ADCC of these anti-CD20 mAbs, when used at a concentration of 1 *μ*M, which is cytotoxic for CLL cells, in line with our previous observations [17]. Owing to the short duration of the assays, effects of kinase inhibitors on ADCC are more likely due to specific inhibition of signaling emanating from the Fc-*γ*-receptors than due to general cytotoxicity for NK effector cells.

While the impairment of the ADCC of anti-CD20 mAbs by ibrutinib may be partly due to decreased CD20 expression on the surface of CLL cells [23], we observed that the enhanced ADCC of Fc- and glycoengineered obinutuzumab was less disturbed by ibrutinib than that of rituximab, similar to ADCC-induced cell lysis as well as NK cell activation and degranulation as surrogate markers in autologous systems [24]. Also with the second-generation of irreversible BTK inhibitors, the ADCC of obinutuzumab against CLL cells was less impaired than that of rituximab, in agreement with reports about the interference of acalabrutinib or tirabrutinib with the ADCC of anti-CD20 mAbs against Mec1 or SUDHL-4 cells, respectively [25]. The different capacity of ibrutinib and acalabrutinib for interfering with the ADCC of anti-CD20 mAbs became apparent, at inhibitor concentrations of 1 *μ*M (Figure 5(a)) or 10 *μ*M [26] in combinations with rituximab or obinutuzumab, respectively.

In conclusion, NK cell line-based ADCC assays reliably indicated superior induction of ADCC against primary CLL cells by obinutuzumab compared to rituximab and less interference by BTK inhibitors. Since these heterologous assays employ stable effector cell lines and thus avoid variability caused by differences in immune status and genetic background of donor cells, they afford robust and sensitive determinations of ADCC against individual CLL samples in 96-well format. This could be useful for preclinical comparisons of combinations of mAbs and KI.
